# Mucormycosis causing massive lower gastrointestinal bleeding: a case report

**DOI:** 10.1186/s12876-021-01846-x

**Published:** 2021-07-02

**Authors:** Ting-Hsuan Chiang, Yi-Wei Lee, Jui-Hsiang Tan, Chih-Chin Kao, Chun-Chao Chang, Kuan-Chieh Fang

**Affiliations:** 1 School of Medicine, College of Medicine, Taipei Medical University, Taipei, Taiwan, ROC; 2 Division of Gastroenterology, Internal Medicine, Taipei Medical University Hospital, No. 252, Wuxing Street, Taipei, Taiwan, ROC; 3 Division of Gastroenterology and Hepatology, Department of Internal Medicine, School of Medicine, College of Medicine, Taipei Medical University, Taipei, Taiwan, ROC

**Keywords:** Mucormycosis, Hematochezia, Diabetic ketoacidosis, Opportunistic infection, Case report

## Abstract

**Background:**

Lower gastrointestinal bleeding (LGIB) is very common in the hospital setting. Most bleedings stop spontaneously, but rare infectious causes of LGIB may lead to rapid and serious complications if left untreated and are sometimes very difficult to diagnose preoperatively.

**Case presentation:**

We described a young man with poorly controlled Type I diabetes mellitus and chronic alcohol abuse who presented with acute altered mental status. During his hospitalization for treatment of diabetic ketoacidosis, acute renal failure, and sepsis, he suddenly developed massive hematochezia of 1500 mL. Colonoscopy was performed and a deep ulcer covered with mucus with peripheral elevation was noted at the transverse colon. Biopsy of the ulcer later revealed nonpigmented, wide (5–20 µm in diameter), thin-walled, ribbon-like hyphae with few septations and right-angle branching suggestive of mucormycosis demonstrated by Periodic acid–Schiff stain. He received 2 months of antifungal treatment. Follow up colonoscopy post-treatment was normal with no ulcer visualized.

**Conclusions:**

Early diagnosis and treatment of gastrointestinal (GI) mucormycosis infection is critical but can be challenging, especially in the setting of massive hematochezia. Therefore, clinical awareness for immunocompromised patients and prompt antifungal prophylaxis in cases with high suspicion of infection are essential.

## Background

Lower gastrointestinal bleeding (LGIB) presenting as hematochezia is very common in the hospital setting. Most cases of LGIB stop spontaneously, but acute severe bleeding can be life-threatening, especially in patients with comorbidities. To improve diagnostic and therapeutic yields, early colonoscopy is recommended for patients with acute LGIB [1].

The most common etiologies of LGIB include diverticulosis, angiodysplasia, colorectal neoplasms, hemorrhoids, and rectal ulcer. Other causes such as inflammatory bowel disease and proctitis are also important differential diagnoses. In immunocompromised patients, opportunistic infections of the GI tract resulting in massive LGIB have been reported in previous literature. These include viral infections such as cytomegalovirus colitis [2], bacterial infections such as tuberculosis [3], and fungal infections such as aspergillosis [4] and mucormycosis [5]. Among these infections, mucormycosis causing LGIB has been the least reported.

Presentations of intestinal mucormycosis reported in literature have been non-specific, ranging from afebrile abdominal pain to severe intestinal perforation [6]. In the few reported cases of mucormycosis presenting as severe LGIB, almost all patients died despite treatment [5, 7]. Delayed diagnosis was often identified as the fundamental reason for mortality [8]. We described a case of GI mucormycosis presenting as massive hematochezia in an immunocompromised patient with diabetic ketoacidosis (DKA). Prompt diagnosis with colonoscopy and antifungal treatment resulted in satisfactory outcome.

## Case presentation

A 28-year-old man was brought to the Emergency Department (ED) by his father due to acute altered mental status and shortness of breath since earlier that morning. His medical history was significant for poorly controlled Type I Diabetes Mellitus with multiple hospitalizations due to diabetes ketoacidosis (DKA), severe chronic alcohol abuse for 8 years, and chronic pancreatitis. He was also diagnosed with nasopharyngeal carcinoma at age 18, for which he underwent concurrent chemoradiotherapy. According to his father, he had not eaten food since the night prior but consumed large amounts of alcohol. Upon arrival at the ED, the patient was stuporous, tachypneic at 26 breaths per minute, blood pressure 134/109 mmHg, heart rate 85 beats per minute, and afebrile. Initial blood work showed glucose of 420 mg/dL, high levels of alcohol (222.7 mg/dL), metabolic acidosis, elevated ketones (5.4 mmol/L), pancytopenia (WBC: 1.03 × 10^3^/uL, Hemoglobin: 9.9 g/dL, Platelet count: 32 × 10^3^/uL) prolonged prothrombin time (19.8 s) and activated partial prothrombin time (> 180 s), hyperammonemia (876 ug/dL), elevated lactate (19.9 mmol/L), elevated liver enzymes (AST 3112 U/L, ALT 328 U/L, Gamma-glutamyl-transferase 1100 U/L), elevated creatinine (2.8 mg/dL) and Blood urea nitrogen (22.0 mg/dL). Initial treatment for his DKA state was given at the ED. However, 3 h later, the patient developed hypotension (BP = 82/42). Norepinephrine was given and the patient was subsequently admitted to the Intensive Care Unit (ICU) for further workup and treatment.

At the ICU, the patient was intubated due to hypoxemic respiratory failure. He underwent continuous renal replacement therapy owing to acute kidney injury and severe metabolic acidosis. Empiric antibiotics were prescribed because of suspected sepsis (Sequential Organ Failure Assessment Score: 9). His blood culture further confirmed growth of Pseudomonas Aeruginosa and Acinetobacter Baumannii. Antibiotics were adjusted according to susceptibility. Additional blood work revealed negative HBV, HCV, and HIV antibodies. His condition gradually improved and was successfully extubated on day 10.

**Fig. 1:**
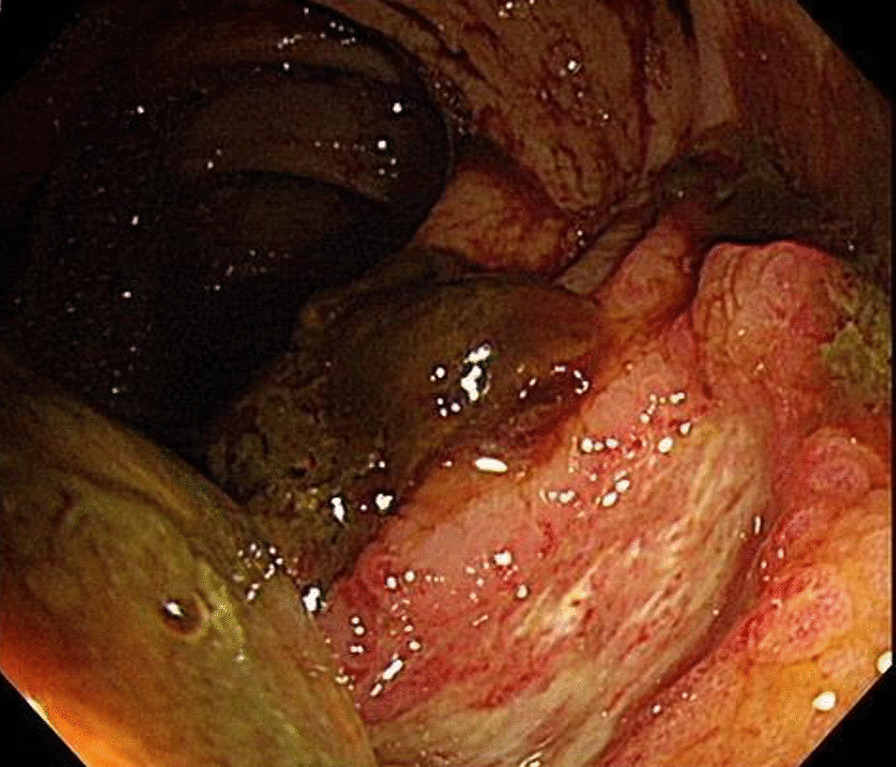
A deep ulcer covered with mucus with peripheral elevation was noted at the transverse colon

**Fig. 2:**
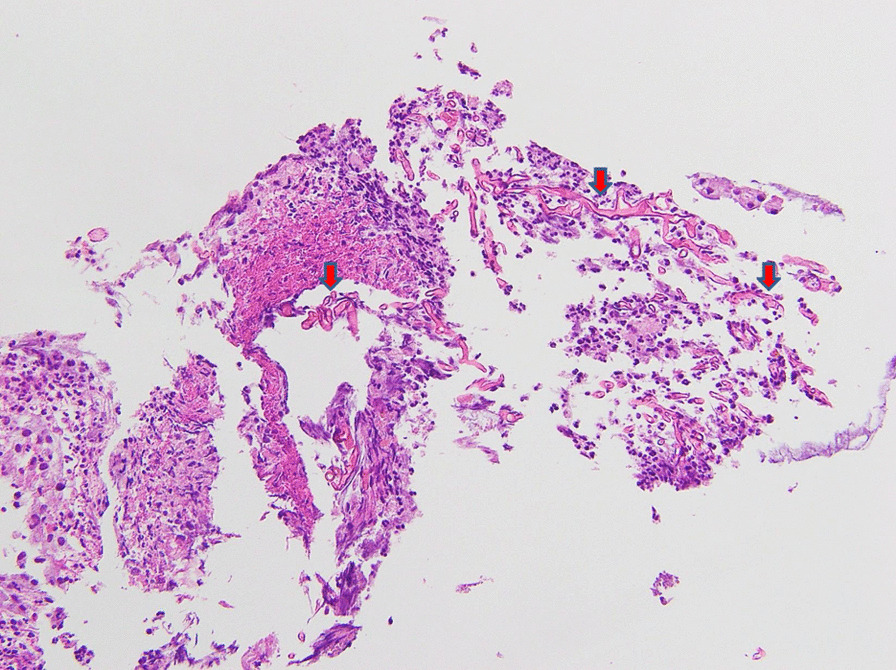
Nonpigmented, wide (5–20 µm in diameter), thin-walled, ribbon-like hyphae with few septations and right-angle branching suggestive of mucormycosis was demonstrated by PAS stain

However, on day 11 of admission, massive hematochezia of 1500 mL was noted, and his hemoglobin dropped from 9.5 to 8.2 g/dL. Blood transfusion, vitamin K, and tranexamic acid were immediately administered. Being hemodynamically stable the next day, the patient underwent colonoscopy. During the procedure, other than blood clots and stool visualized along the proceeding scope, a deep ulcer covered with mucus with peripheral elevation was noted at the transverse colon (Fig. 1). A biopsy was done and the specimen was sent to pathology. Histopathology of the biopsy revealed nonpigmented, wide (5–20 µm in diameter), thin-walled, ribbon-like hyphae with few septations and right-angle branching suggestive of mucormycosis demonstrated by Periodic acid–Schiff (PAS) stain (Fig. 2). Blood cultures taken at that time were negative and upper endoscopy did not reveal similar lesions.

**Fig. 3:**
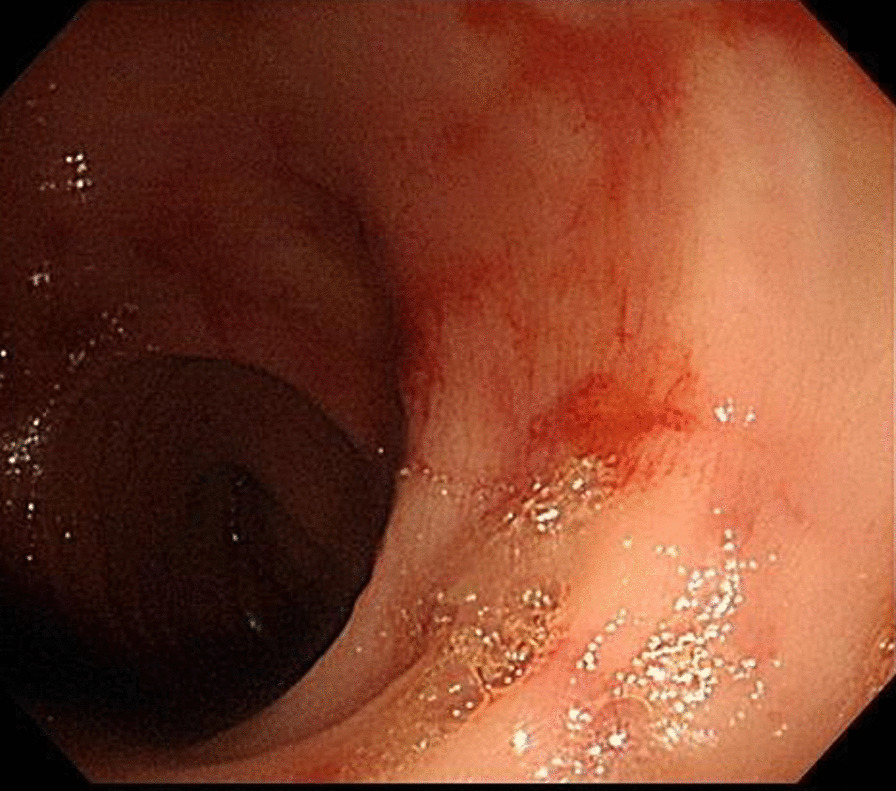
No lesions were visualized upon follow-up colonoscopy post-treatment

Upon receiving the pathology report 5 days after the colonoscopy, the patient was started on oral Posaconazole therapy. No hematochezia was observed after starting the treatment and his c-reactive protein levels continued to decline (from 3.2 to 0.16). The patient’s condition gradually stabilized and was subsequently stepped down from the ICU to the ward and discharged 2 weeks later. He received a total of 2 months of Posaconazole therapy. One month post treatment, a follow up colonoscopy did not reveal any lesions (Fig. 3).

## Discussion

One rare cause of lower GI bleeding is GI mucormycosis. Mucormycosis is an opportunistic infection caused by fungi in the order of Mucorales [9]. The low virulence of these organisms makes disease rare among immunocompetent individuals [10]. However, prognosis among infected individuals is extremely poor, and mortality has been found to be associated with the site of infection [9]. Most infected patients have underlying factors of compromised immunity, such as immunosuppression because of organ transplantation, malignancy, persistent neutropenia, poorly controlled diabetes mellitus and systemic corticosteroid therapy [7, 11].

Mucormycosis can manifest as different clinical syndromes: rhino-orbito-cerebral, pulmonary, cutaneous, gastrointestinal, disseminated, and uncommon presentations. Among these manifestations, gastrointestinal mucormoycosis is one of the rarest, making up only around 7% of all cases [10]. Disease of the gastrointestinal tract is usually acquired through ingestion of contaminated food infection of an implanted device. Within the gastrointestinal tract, the stomach is the most common sight of infection, followed by the colon. Mortality of GI mucormyocosis has been reported to be as high as 85% [12]. Patients often present with nonspecific symptoms such as abdominal pain, GI bleeding, change in bowel habits, and fever [9]. Interestingly, the majority of patients present with abdominal pain in the absence of fever [13]. Nonspecific clinical presentation makes the diagnosis difficult and often delayed, and therefore contributes to the high mortality rate. It is estimated that less than half of the diagnosis of mucormycosis are made antepartum [11].

Definite diagnosis of mucormycosis is made by histopathological evidence of fungal invasion of tissue. Unlike aspergillosis, there is no reliable serological test for mucormycosis. In the case of GI mucormycosis, undergoing invasive endoscopy or even surgery is usually inevitable for diagnosis. A dark appearing ulcer with sharp demarcated edges apparently related to necrosis and thrombosis in adjacent vessels is representative of a typical GI lesion [11]. Endoscopic finding of a small, mushroom-like green mass with a small base attached to the colon wall has also been described in previous studies [14]. Extended lesions can cause perforation, bleeding, and obstruction.

With the widespread availability of endoscopic techniques, early clinical suspicion and diagnosis as well as effective treatment are critical in reducing mortality. However, many patients with severe acute LGIB are unable to tolerate rapid colon preparation for diagnostic colonoscopy, making localization of lesions challenging due to poor visualization. Therefore, in immunocompromised patients such as those with DKA, pharmacological immunosuppression because of organ transplantation, or diseases requiring immunosuppressive medications, it is important to consider the possibility of GI mucormycosis. In the absence of other immediately identifiable etiologies, empiric antifungal treatment should be initiated without delay for clinically suspicious cases of acute severe LGIB in patients who are unable to undergo timely definite diagnosis via colonoscopy.

It is advised that treatment of mucormycosis include urgent surgical resection of infected area whenever feasible parallel to systemic antifungal therapy to prevent disease dissemination [7, 8]. Liposomal amphotericin B is the recommended first line treatment for mucormycosis. Posaconazole has generally been recommended as maintenance and salvage therapy. However, Song et al. reported a higher survival rate in renal transplant patients with mucormycosis treated with Posaconazole compared to Amphotericin B [15]. The European Confederation of Medical Mycology 2019 guideline proposes the use of Posaconazole as first line treatment with moderate strength, especially in case of renal failure like our patient [16]. Reversal of underlying problems is also fundamental to improve treatment outcome [7, 11]. A study by Gebremariam et al. highlighted the importance of correcting acidemia in patients with ketoacidosis [17]. For our patient, proper control of his diabetes mellitus and treatment of alcoholism are of great importance.

## Conclusion

Mucormycosis is a life-threatening fungal infection caused by Mucorales, primarily affecting the immunocompromised hosts. A rare clinical presentation of mucormycosis of the GI tract is hematochezia. Early diagnosis and treatment of mucormycosis infection is critical but can be challenging, especially in the setting of massive hematochezia where adequate bowel preparation for diagnostic colonoscopy cannot be achieved. Therefore, colonic opportunistic infections such as mucormycosis should be a differential diagnosis for massive GI bleeding in immunocompromised patients. In cases with high clinical suspicion, prompt empiric treatment of possible fungal infection is critical.

## Data Availability

All data generated during this study are included in this published article.

## Abbreviations

LGIB: Lower gastrointestinal bleeding
GI: Gastrointestinal
ED: Emergency department
DKA: Diabetic ketoacidosis
AST: Aspartate transaminase
ALT: Alanine aminotransferase
ICU: Intensive care unit
HBV: Hepatitis B virus
HCV: Hepatitis C virus
HIV: Human immunodeficiency virus
PAS: Periodic acid–Schiff
